# Two-Scale Multimodal Medical Image Fusion Based on Structure Preservation

**DOI:** 10.3389/fncom.2021.803724

**Published:** 2022-01-31

**Authors:** Shuaiqi Liu, Mingwang Wang, Lu Yin, Xiuming Sun, Yu-Dong Zhang, Jie Zhao

**Affiliations:** ^1^College of Electronic and Information Engineering, Hebei University, Baoding, China; ^2^Machine Vision Technological Innovation Center of Hebei, Baoding, China; ^3^School of Mathematics and Information Science, Zhangjiakou University, Zhangjiakou, China; ^4^School of Computing and Mathematics, University of Leicester, Leicester, United Kingdom

**Keywords:** medical image fusion, scale decomposition, structure preservation, bilateral filter, CNN

## Abstract

Medical image fusion has an indispensable value in the medical field. Taking advantage of structure-preserving filter and deep learning, a structure preservation-based two-scale multimodal medical image fusion algorithm is proposed. First, we used a two-scale decomposition method to decompose source images into base layer components and detail layer components. Second, we adopted a fusion method based on the iterative joint bilateral filter to fuse the base layer components. Third, a convolutional neural network and local similarity of images are used to fuse the components of the detail layer. At the last, the final fused result is got by using two-scale image reconstruction. The contrast experiments display that our algorithm has better fusion results than the state-of-the-art medical image fusion algorithms.

## Introduction

Medical imaging technology has developed rapidly, and medical images of various modalities have significant applications in clinical diagnosis and disease analysis (Wang S. et al., 2020; Li et al., 2021). There are some common medical imaging technologies, such as CT, MRI, PET, and single-photon emission computed tomography (SPECT). Due to different imaging technologies, various kinds of medical images contain complementary characteristics. For the same organ or the same tissue of the human body, medical images with different imaging technologies reflect various pathological characteristics. CT can well show dense structures, such as bones, while MRI has a higher spatial resolution for soft tissues. PET and SPECT are more conducive to display functional information of different tissues of the human body. However, limited by the equipment, the quality of medical images is often unsatisfying. Two or more medical images are merged into one image with comprehensive features by fusion methods for the sake of improving the accuracy of clinical diagnosis (Li et al., 2017; Amala Rani and Lalithakumari, 2019; Huang et al., 2020). Medical image fusion aims at extracting more useful information of input images, improving the application of medical images, and helping doctors understand image contents better. Therefore, the study of medical image fusion is significant (Tirupal et al., 2020).

The existing image fusion methods include pixel-level image fusion methods, feature-level image fusion methods, and decision-level image fusion methods (Dolly and Nisa, 2019; Zhao et al., 2021). The pixel-level fusion methods fuse multiple registered images and preserve the maximum amount of information. Feature-level fusion algorithms extract different features from input images, such as textures and details, and then merge all the features into one image. Feature-level image fusion has lower requirements for image registration so it will lose part of source images’ detailed information. Decision-level fusion is performed by the high-level abstracted information, so the detailed information will be lost severely (Kim et al., 2016; Liu Z. et al., 2019). In conclusion, pixel-level fusion algorithms are the simplest methods with the lowest computation cost. Therefore, we propose a pixel-level medical image fusion algorithm.

In general, pixel-level image fusion methods contain spatial domain methods and transform domain methods (Yu and Chen, 2020). In spatial domain, the fusion operation is performed on pixels (Ashwanth and Swamy, 2020; Zhu et al., 2020). Spatial domain fusion algorithms include principal component analysis (PCA)-based image fusion method (Benjamin and Jayasree, 2018), guided filtering (GFF)-based image fusion method (Li et al., 2013), improved sum-modified-Laplacian (ISML)-based image fusion algorithm (Liu et al., 2015a), nuclear norm minimization (NNM)-based image fusion algorithm (Liu et al., 2015b), convolutional neural network (CNN)-based image fusion algorithm (Liu et al., 2017b), and so on. In transform domain image fusion algorithms, source images are first decomposed into low- and high-frequency components through multi-scale transformations. And then appropriate fusion rules are used to fuse different components. Finally, the final fused result can be obtained through inverse transformation. At present, scholars around the world have proposed many image fusion methods in the transform domain (Mao et al., 2020), such as the wavelet-based image fusion methods proposed in Sumir and Gahan (2018), Panigrahy et al. (2020b), and Rahmani et al. (2020), the image fusion algorithms using non-subsampled contourlet transform (NSCT) proposed in Das and Kundu (2012), Zhu et al. (2019), Liu J. et al. (2020), and Panigrahy et al. (2020a), and the image fusion algorithms using non-subsampled shearlet transform (NSST) (Liu et al., 2017a; Ganasala and Prasad, 2020; Panigrahy et al., 2020c). These algorithms have achieved good fusion effects. However, traditional transform domain image fusion algorithms need to perform frequency decomposition and synthesis during fusion, which often have problems, such as fused image distortion and structural information loss of the source images (Zeng et al., 2020). However, in spatial domain, image fusion often needs to block the images during fusion, which often produces serious blocking effects. To maintain the structural and detailed information, Zhu et al. (2018) proposed an image fusion method based on image cartoon-texture decomposition and sparse representation. In recent years, many structure-preserving filters have appeared (Tomasi and Manduchi, 1998; Farbman et al., 2008; Xu et al., 2011; He et al., 2019). These filters can well maintain the structural information in the images while smoothing textures, which is beneficial to restore the structures and neighborhood details of source images. Therefore, structure-preserving filters are increasingly used in image fusion. The proposed algorithm also applies a structure-preserving filter to overcome block effects and maintain structural information. Recently, deep learning-based fusion algorithms became a hotspot (Tomasi and Manduchi, 1998; Zhu et al., 2018). A multi-focus image fusion method by using CNN is proposed in Farbman et al. (2008), which regards the generation process of the information feature focus map as a classification problem, and the fusion rule can be regarded as the classifiers used in general classification tasks. Liu S. et al. (2019) proposed a residual network-based multi-focus image fusion method. Subsequently, deep learning has also been applied in medical image fusion. Liu et al. (2015b) also proposed a CNN-based medical image fusion algorithm, which achieved satisfying fusion results. These algorithms can effectively integrate the design of fusion rules and the generation of a decision map, greatly simplifying the steps of image fusion. However, for the sake of obtaining better image fusion results, deep learning-based medical image fusion algorithms need to train a great number of samples. In the training of the network, the training data sets should have ground-true images, but it is not easy to obtain ground-true images of medical images.

Recently, deep learning-based fusion algorithms became a hotspot (Wang et al., 2021a,b). A multi-focus image fusion method by using CNN is proposed in Liu et al. (2017c), which regards the generation process of the information feature focus map as a classification problem, and the fusion rule can be regarded as the classifiers used in general classification tasks. Liu S. et al. (2019) proposed a residual network-based multi-focus image fusion method. Subsequently, deep learning has also been applied in medical image fusion. Liu et al. (2017b) proposed a CNN-based medical image fusion algorithm, which achieved satisfying fusion results. Wang K. et al. (2020) proposed a CNN-based multi-modality medical image fusion algorithm. This algorithm uses the Siamese convolutional network to generate the weight map. The source images are decomposed by contrast pyramid and then fused based on the weight map. These algorithms can effectively integrate the design of fusion rules and the generation of a decision map, greatly simplifying the steps of image fusion. However, for the sake of obtaining better image fusion results, deep learning-based medical image fusion algorithms need to train a great number of samples. In the training of the network, the training data sets should have ground-true images, but it is not easy to obtain ground-true images of medical images.

For the sake of solving the above-mentioned problems of image fusion algorithms, we proposed a two-scale medical image fusion algorithm based on structure preservation. Aiming at different imaging modes of multimodal medical images, a two-scale decomposition operation is adopted to decompose source images into base layer components and detail layer components. In this algorithm, we used different strategies to fuse different components, making the fusion process more comfortable for the human visual system. In the fusion of the base layer components, an iterative joint bilateral filter is applied to improve the decision map, and the weighted sum strategy is used to fuse the base layer components in the spatial domain. In the proposed algorithm, the CNN is adopted to fuse components of the detail layer to produce an image fusion weight map. The components of the detail layer are then fused by employing a multi-scale method and a fusion strategy based on local similarity. In the proposed algorithm, we made full use of the structure-preserving property of the iterative bilateral filter. In addition, the application of CNN also improves the performance of medical image fusion. Compared with previous algorithms, the main contribution of our method consists of three points. (1) We use a two-scale decomposition method to decompose source images into base layer components and detail layer components, which can make full use of scale information. (2) For the sake of retaining more information, a fusion strategy based on an iterative joint bilateral filter is adopted to fuse base layer components. (3) A new fusion rule based on CNN and local similarity was applied to the detail layer to maintain more detailed information.

The rest of this article is as follows. Section “Related Works” introduces the related works. Section “The Proposed Algorithm” describes the proposed fusion method and its implementation steps. Section “Experiments and Discussion” shows the experimental results and analysis of this algorithm. Finally, in Section “Conclusion” we conclude our fusion method.

## Related Works

### Two-Scale Image Decomposition

For a given source image *I*, it can be decomposed into the base layer component *I*^*b*^ and the detailed layer component *I*^*d*^. *I*^*b*^ can be obtained through the following optimization problem:


(1) $$\arg\,\min\,{||I-I_{b}||}_{F}^{2}+\eta \,\left({||g_{x}*I^{b}||}_{F}^{2}+{||g_{y}*I^{b}||}_{F}^{2}\right)$$

where *g*_*x*_ = [−1 1] and *g*_*y*_ = [−1 1]^*T*^ are horizontal and vertical gradient operators, respectively. The regularization parameter η is set to 5. The optimization problem is a Tikhonov regularization problem, and it can be resolved efficiently by fast Fourier transform (FFT). By subtracting the base layer component from the source image, we can get the detail layer component *I*^*d*^, that is


(2) $$I^{d}=I-I^{b}$$

### Iterative Joint Bilateral Filter

A bilateral filter is non-linear and it can achieve edge preservation during denoising (Tomasi and Manduchi, 1998). In bilateral filtering, the filtered pixel value of a point depends on the neighborhood pixels. Furthermore, the weights of the neighborhood pixels are determined by the distance and similarity of the two pixels.

The bilateral filter can be represented as


(3) $$J_{p}=\frac{1}{k_{p}}\,\sum_{q\in \Omega }\exp\left(-\frac{{||\text{p–q}||}^{2}}{2\,\sigma_{s}^{2}}-\frac{{||I_{p}-I_{q}||}^{2}}{2\,\sigma_{r}^{2}}\right)\,I_{q}$$

where *I* is the input image. *p* and *q* are the coordinates of the pixels. *I*_*p*_ and *I*_*q*_ denote the pixel values of the corresponding positions. Ω is a sliding window centered on *p*. *J*_*p*_ is the output. σ_*s*_ and σ_*r*_ control the weight of spatial domain and range domain, respectively. $k_{p}=\sum_{q\in \Omega }\exp\left(-\frac{{||\text{p–q}||}^{2}}{2\,\sigma_{s}^{2}}-\frac{{||I_{p}-I_{q}||}^{2}}{2\,\sigma_{r}^{2}}\right)$ is a normalization.

The weight of the bilateral filter is unstable in practical applications, so some artificial textures will appear near the edges. For the sake of improving the stability of weight, a joint bilateral filter (Zhan et al., 2019) is introduced. It can deal with the problem of the stability of weight by introducing a guide image as the basis for calculating the range weight.

In this article, the iterative joint bilateral filter is used to refine edges. In filtering operation, *J*^1^ represents the initial input guide image and *J*^t+1^ denotes the output of the *t*-th iteration, that is


(4) $$J_{p}^{t+1}=\frac{1}{k_{p}}\,\sum_{q\in \Omega }\exp\left(-\frac{{||\text{p–q}||}^{2}}{2\,\sigma_{s}^{2}}-\frac{{||J_{p}^{t}-J_{q}^{t}||}^{2}}{2\,\sigma_{r}^{2}}\right)\,I_{q}$$

where *k*_*p*_ is used to normalize,


(5) $$k_{p}=\sum_{q\in \Omega }\exp\left(-\frac{{||\text{p–q}||}^{2}}{2\,\sigma_{s}^{2}}-\frac{{||J_{p}^{t}-J_{q}^{t}||}^{2}}{2\,\sigma_{r}^{2}}\right)$$

where σ_*s*_ is set to 8 and σ_*r*_ is set to 0.2.

### Convolutional Neural Network

Convolutional neural network is widely used in behavior cognition, pose estimation, object recognition, neural network conversion, and natural language processing. It is one of the most commonly used models in deep learning. It is composed of input layer, hidden layer, and output layer. The hidden layer contains convolutional layer, pooling layer, and fully connected layer. There are three structural characteristics in the CNN model, which are local receptive field, weight sharing, and pooling. The local receptive field represents the connection between a single neuron and the neurons in the corresponding region of the previous layer. As the number of layers deepens, the corresponding receptive field of a single neuron in each layer increases in the original image, and the information expressed becomes more and more abstract. Weight sharing means that the weight of the convolution kernel of feature mapping is spatially invariant. The receptive field and weight sharing decrease the number of parameters for neural network training. Pooling is also called down-sampling, which can reduce data dimensions. The core idea of CNN is to combine these three structures to obtain the deep features of images.

The calculation formula of the convolution kernel is


(6) $$y_{j}^{l}=f\,\left(\sum_{i\in M_{j}}y_{i}^{l-1}*k_{i\,j}^{l}+b_{j}^{l}\right)$$

where *M*_*j*_ represents the feature map from the input. $k_{i\,j}^{l}$ represents the convolution kernel connected to the *i*-th feature map of the previous layer. * represents convolution operation. $b_{j}^{l}$ represents the bias. *f*(⋅) represents the activation function.

In CNN-based image fusion algorithms, the generation of a decision map can usually be considered as a classification problem. Specifically, the decision map can be acquired through activity level measurement. Measuring the activity level of a source image is closely related to feature extraction. The higher the activity level means that the image block is clearer. Image fusion is similar to classification tasks. Therefore, the fusion rule in this process is equal to the classifiers in image classification. According to these ideas, a CNN-based multi-focus image fusion algorithm is proposed in Liu et al. (2017c). The advantage of this algorithm is that it solves the difficulties of manually designing activity level measurement and the fusion rule. Therefore, the CNN-based image fusion algorithm solves the problem of fusion by designing the CNN model. By training a large number of image data to obtain the CNN model, it is more effective than manually designing the fusion rule. In Liu et al. (2017b), combining with the CNN model, Liu et al. used a multi-scale method and a local similarity-based fusion rule to obtain a high-quality fusion result. We applied this method to fuse detail components in our fusion algorithm.

## The Proposed Algorithm

**FIGURE 1:**
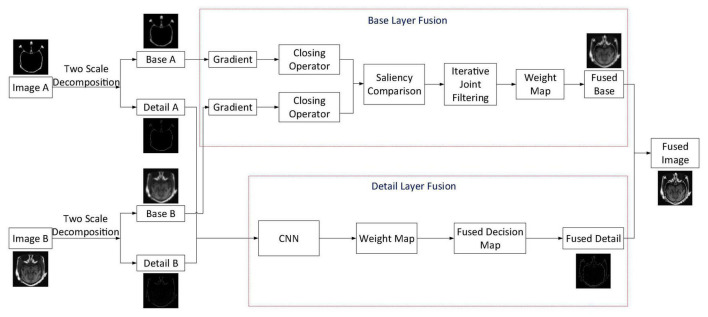
The framework of the fusion algorithm.

Aiming at obtaining a satisfying medical image fusion result, we proposed a two-scale medical image fusion algorithm based on structure preservation. The framework of this fusion method is shown in Figure 1. First, through the method in Section “Two-Scale Image Decomposition,” we decompose source images into base and detail layers. After that, these scale components are fused by appropriate fusion methods. Finally, the fused image is obtained through scale reconstruction.

For ease of description, we suppose that the two source images are *I*_*A*_ and *I*_*B*_, and in this article, we have given the fusion steps of two images only. The process of multiple images fusion is similar to the fusion of two images. Traditional multi-scale decomposition methods need more than two scales to get a satisfactory fusion image. In the proposed algorithm, we decomposed the source images by using the two-scale decomposition method in Liu Y. et al. (2016). Equations 1, 2 are employed to decompose source images *I*_*A*_ and *I*_*B*_ into base layer components $I_{A}^{b}$, $I_{B}^{b}$ and detail layer components $I_{A}^{d}$, $I_{B}^{d}$. The following are descriptions of the fusion rules of base and detail layer components.

### Base Layer Fusion

First, a local structure preservation filter is used to smooth the base layer components $I_{A}^{b}$ and $I_{B}^{b}$. This filter is defined as


(7) $$S_{p}=\mu_{k}+\frac{\sigma_{k}^{2}}{\sigma_{k}^{2}+\sigma_{d}^{2}}\,\left(I_{p}-\mu_{k}\right)$$

where *p* represents the pixel position. μ_*k*_ and $\sigma_{k}^{2}$ are the mean and variance of the image pixel values in a sliding window Ω_*k*_ with radius *r*, respectively. The size of the window Ω_*k*_ is (2*r* + 1)×(2*r* + 1). $\sigma_{d}^{2}$ is a constant. The mean value μ_*k*_ represents the average smoothing intensity of the filter, and its variance $\sigma_{k}^{2}$ reflects and measures the changes in local sharpness of the image. In Eq. 7, if $\sigma_{k}^{2}\gg \sigma_{d}^{2}$, there is *S*_*p*_ = *I*_*p*_ for pixel *p*; if $\sigma_{k}^{2}\ll \sigma_{d}^{2}$, then *S*_*p*_ = μ_*k*_. Therefore, we set $\sigma_{d}^{2}$ as the minimum variance of the source image to achieve smoothing of tiny pixels. In this study, after many experiments, we found that our algorithm works best when *r* is set to 3 and $\sigma_{d}^{2}$ is set to 0.01.

In this article, a two-stage gradient is adopted to describe the salient area of the image. The gradient image *G*_*p*_ can be defined as


(8) $$G_{p}={\left(S_{p}⊗L\right)}^{2}$$

where ⊗ represents convolution. *L* is the gradient detection template, In this article, for getting more detailed information, we introduced a gradient detection template in Zhan et al. (2019), that is


(9) $$L=\frac{1}{6}\,\left[\begin{array}{ccc} 1 & 4 & 1\\ 4 & -20 & 4\\ 1 & 4 & 1 \end{array}\right]$$

Since holes may be generated in homogeneous regions when performing gradient operation on the image, we adopted morphological closure operation to fill the image holes in this article, that is


(10) $${\hat{M}}_{p}=1-\left(1-G_{p}⊗E\right)⊗E$$

where *E* denotes the sliding square structure element with radius _*r*_.

By using Eq. 10, we can obtain the structural gradients ${\hat{M}}_{p}^{A}$ and ${\hat{M}}_{p}^{B}$ of each base layer component. Then, the weight map is constructed by using the strategy of taking the larger absolute value. For the image value at the pixel *p* at the same position, the obtained binary weight map *B*_*p*_ can be calculated as follows.


(11) $$B_{p}=\left\{ \begin{array}{cc} 1 & {\hat{M}}_{p}^{A}\geq {\hat{M}}_{p}^{B}\\ 0 & {\hat{M}}_{p}^{A}<{\hat{M}}_{p}^{B} \end{array}\right.$$

*B*_*p*_ is obtained by selecting the saliency structure between $I_{A}^{b}$ and $I_{A}^{b}$ during image fusion. For the sake of making the fused image have more spatial continuity, we can get the basic weight map *W*_*p*_ by performing average filtering on *B*_*p*_ with a window size of 5 × 5. To make the image look more natural, the iterative joint bilateral filter is employed to transfer the edge information in source images into the weight map. The final decision map *W*_*p*_ is obtained through three iterations, that is


(12) $$W_{p}=b\,i\,l\,t\,e\,r\,a\,l\,\_\,f\,i\,l\,t\,e\,r\,\left(I_{p},W_{p},\sigma_{s},\sigma_{r}\right)$$

Finally, the weighted sum fusion method is used to acquire the fused base layer component.


(13) $$I_{F}^{b}=I_{A}^{b}\,W_{p}+I_{B}^{b}\,\left(1-W_{p}\right)$$

### Detail Layer Fusion

**FIGURE 2:**
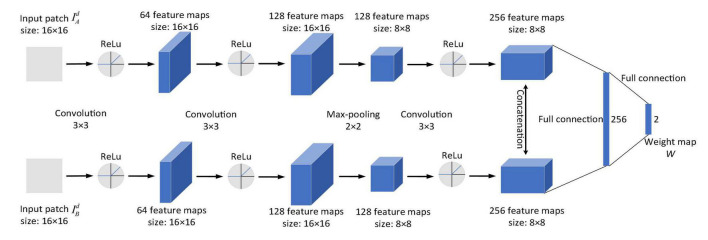
The structure of convolutional neural network.

Combined with CNN, the Laplacian pyramid and the fusion strategy based on local similarity are adopted for the fusion of detail layer components in the proposed algorithm. The CNN structure adopted is shown in Figure 2.

It has two identical branches and each branch has three convolutional layers and a max-pooling layer. In each convolutional layer, an ReLu is added for non-linear mapping, which has fast convergence speed and a simple gradient operation. The size of the neuron’s receptive field depends on the size of the convolution kernel. Choosing an appropriate convolution kernel size is significant for CNN. If the convolution kernel is too small, local features cannot be extracted effectively; and if the kernel size is too large, the information representation capability is reduced and the image cannot be represented well. Therefore, during net training, the kernel size and step of each convolutional layer are set to 3 × 3 and 1, respectively. And the scale factor and span of the max-pooling layer are set to 2 × 2 and 2, respectively. The 256 feature maps are connected to a 256-dimensional feature vector through a fully connected layer. The output is a fully concatenated two-dimensional vector of the 256-dimensional feature vector.

In this algorithm, the two detail layer components $I_{A}^{d}$ and $I_{B}^{d}$ are input into the two branches of the CNN to generate a fusion weight map *W*. Then, Laplacian pyramid decomposition is performed on each detail layer component, which is denoted by *L*{*A*}^*l*^ and *L*{*B*}^*l*^, respectively (_*l*_ denotes the decomposition scale). The weight map *W* is decomposed by Gaussian pyramid and expressed by *G*{*W*}^*l*^.

The local energy maps of *L*{*A*}^*l*^ and *L*{*B*}^*l*^ are calculated as


(14) $$\left\{ \begin{array}{c} E_{A}^{l}\,\left(x,y\right)=\sum_{m}\sum_{n}L\,{\left\{A\right\}}^{l}\,{\left(x+m,y+n\right)}^{2}\\ E_{B}^{l}\,\left(x,y\right)=\sum_{m}\sum_{n}L\,{\left\{B\right\}}^{l}\,{\left(x+m,y+n\right)}^{2} \end{array}\right.$$

Then, we calculate the local similarity measurement of *L*{*A*}^*l*^ and *L*{*B*}^*l*^, that is


(15) $$M^{l}\,\left(x,y\right)=\frac{2\,\sum_{m}\sum_{n}L\,{\left\{A\right\}}^{l}\,\left(x+m,y+n\right)\,L\,{\left\{B\right\}}^{l}\,\left(x+m,y+n\right)}{E_{A}^{l}\,\left(x,y\right)+E_{B}^{l}\,\left(x,y\right)}$$

From Eq. 15, we can know that the range of *M*^*l*^(*x, y*) is [−1,1]. The closer the value is to 1, the higher the local similarity of *L*{*A*}*^l^* and *L*{*B*}*^l^* is. The threshold *t* is set to fuse pixels with different similarities using different fusion rules. If *M*^*l*^(*x, y*) ≥ *t*, *L*{*A*}*^l^* and *L*{*B*}*^l^* are fused through the weighted average fusion method, that is


(16) $$L\,{\left\{F\right\}}^{l}\,\left(x,y\right)=G\,{\left\{W\right\}}^{l}\,\left(x,y\right)\cdot L\,{\left\{A\right\}}^{l}\,\left(x,y\right)+\left(1-G\,{\left\{W\right\}}^{l}\,\left(x,y\right)\right)\cdot L\,{\left\{B\right\}}^{l}\,\left(x,y\right)$$

If *M*^*l*^(*x, y*) < *t*, the fusion will be carried out by maximizing the local energy, that is


(17) $$L\,{\left\{F\right\}}^{l}\,\left(x,y\right)=\left\{ \begin{array}{c} \begin{array}{ccc} L\,{\left\{A\right\}}^{l}\,\left(x,y\right), & i\,f & E_{A}^{l}\,\left(x,y\right)\geq E_{B}^{l}\,\left(x,y\right) \end{array}\\ \begin{array}{ccc} L\,{\left\{B\right\}}^{l}\,\left(x,y\right), & i\,f & E_{A}^{l}\,\left(x,y\right)<E_{B}^{l}\,\left(x,y\right) \end{array} \end{array}\right.$$

Ultimately, the fused detail layer component $I_{F}^{d}$ is reconstructed from the Laplacian pyramid *L*{*F*}*^l^*. Then the fused image *I*_*F*_ is reconstructed by fused base layer component $I_{F}^{b}$ and detail layer component $I_{F}^{d}$, that is


(18) $$I_{F}=I_{F}^{b}+I_{F}^{d}$$

## Experiments and Discussion

**FIGURE 3:**
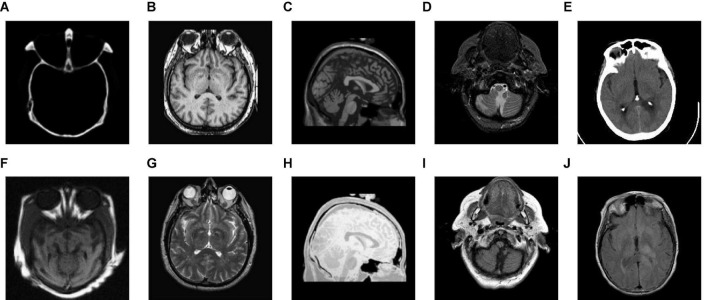
Source image sets for fusion testing. **(A–C,E)** CT images. **(D)** MR-T1 image. **(F–H)** The corresponding MRI images. **(I)** The corresponding MR-T2 image. **(J)** Proton density-weighted MR image.

In the study, five groups of medical images are chosen for testing, to evaluate the performance of the proposed fusion algorithm. These test images are displayed in Figure 3 and are all from http://www.med.harvard.edu/aanlib/home.html. These test image sets contain five groups, among which groups (a), (b), and (c) include three CT images of bones and three MRI images of corresponding soft tissues. Group (d) includes an MR-T1 image and a corresponding MR-T2 image. Group (e) includes a CT image and a proton density-weighted MR image. All experiments are carried out under the computer configuration of 2.9 GHz CPU and 8 GB RAM, MATLAB version of R2018a, and the operating system of Win10 64-bit.

To verify the effectiveness of the proposed algorithm (abbreviated as DWTRP-CNN), we first conducted fusion on the test images and compared the proposed algorithm with commonly used fusion algorithms. These algorithms include (1) the guided filtering based image fusion method proposed in Li et al. (2013) (abbreviated as GFF); (2) the CNN-based medical image fusion method proposed in Liu et al. (2017b) (abbreviated as CNN); (3) an image fusion algorithm by using convolutional sparse morphological component analysis proposed in Liu Y. et al. (2019) (abbreviated as CSMCA); (4) a medical image fusion method based on pulse coupled neural network and improved spatial frequency in NSCT domain proposed in Das and Kundu (2012) (abbreviated as NSCT-PCNN); (5) a multimodal medical image fusion algorithm based on phase consistency and local Laplacian energy in NSCT domain proposed in Zhu et al. (2019) (abbreviated as NSCT-PCLLE); (6) a parameter adaptive pulse coupled neural network-based medical image fusion algorithm in NSST domain proposed in Yin et al. (2019) (abbreviated as NSST-PAPCNN); and (7) the rolling guidance filtering multimodal medical image fusion by combining CNN and nuclear norm minimization proposed in Liu S. et al. (2020) (abbreviated as RGF-CNM). To make it fair, when using the above-mentioned fusion algorithms for testing, the parameter settings of these algorithms have the same parameters in the articles published by the authors.

We also choose six indicators for objective evaluation of the proposed algorithm, namely, Mutual Information (MI) (Qu et al., 2002), QAB/F metric (Xydeas and Petrovic, 2000), Structural Similarity (SSIM) (Wang et al., 2004), Visual Information Fidelity (VIFF) (Han et al., 2013), Universal Image Quality Index (UIQI) (Wang and Bovik, 2002), Piella index (Q, QW, QE) (Piella and Heijmans, 2003), and R${}_{\text{Q}}^{\mathrm{AB}/F}$ metric (Sengupta et al., 2020). MI reflects the useful information that remained in a fused image. The QAB/F index is an objective measure based on edge information and is adopted to assess whether the fused image retains more edge information. SSIM represents the structural similarity between the fused image and the source images. VIFF measures the fidelity of the visual information of the fused image relative to the source images. UIQI measures image distortion through correlation loss, brightness distortion, and contrast distortion. The Piella index comprehensively reflects the similarity of intensity, contrast, and structure between the fused image and the source images. R${}_{\text{Q}}^{\mathrm{AB}/F}$ metric reflects the edge and orientation strengths. For all of these indexes, the higher value means that the performance of the fusion method is better.

**FIGURE 4:**
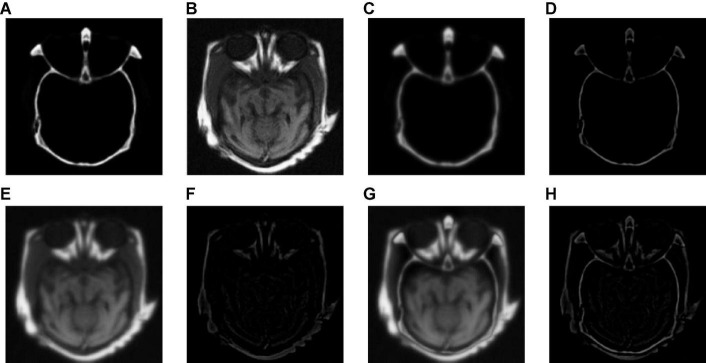
The output images obtained after each step of Group (a). **(A,B)** The source images. **(C)** The base layer component of panel **(A)**. **(D)** The detail layer component of panel **(A)**. **(E)** The base layer component of panel **(B)**. **(F)** The detail layer component of panel **(B)**. **(G)** The fused base layer component. **(H)** The fused detail layer component.

Figure 4 shows the output images obtained after each step of our fusion method applied in Group (a). Figures 4A,B are the source images. Figures 4C,D are the base layer component and the detail layer component of Figure 4A, respectively. Figures 4E,F are the base layer component and the detail layer component of Figure 4B, respectively. Figures 4G,H are the fused base layer component and the fused detail layer component, respectively. It can be seen from Figure 4 that the proposed algorithm can extract the basic information and detailed information of source images and fuse them effectively.

**FIGURE 5:**
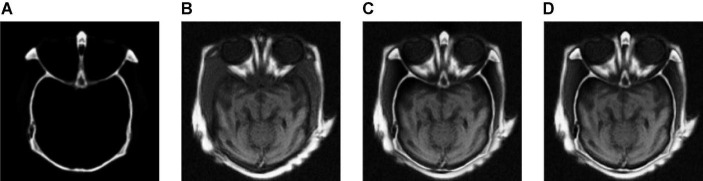
Ablation study of CNN. **(A,B)** The source images.

To verify the effectiveness of CNN for detail layer fusion, we conducted an ablation study on Group (a). Figure 5 shows the fusion results of the ablation study. Figures 5A,B show the source images. Figure 5C is the fusion result without CNN, that is, the detail layers are fused by the method which is the same as the method for fusing base layers. Figure 5D is the fusion image of the proposed algorithm.

Table 1 shows the objective evaluation results of the ablation study. It can be seen from Table 1 that the fusion algorithm with CNN has better objective evaluation results. The ablation study proves the effectiveness of CNN for detail layer fusion.

**TABLE 1:** Objective evaluation results of ablation study.

Method	MI	Q^AB/F^	SSIM	VIFF	UIQI	Q	Q_W_	Q_E_	R${}_{Q}^{A\,B/F}$
Without CNN	3.9219	0.7546	0.4912	0.4600	0.4646	0.8395	0.8071	0.7854	0.3786
CNN	**4.3328**	**0.7869**	**0.4990**	**0.4684**	**0.4770**	**0.8636**	**0.8300**	**0.8185**	**0.3839**

*Abbreviations: MI, mutual information; QAB/F, metric; SSIM, structural similarity; VIFF, visual information fidelity; UIQI, Universal Image Quality Index; Q, QW, QE, Piella index; R${}_{Q}^{A\,B/F}$, metric.*

*The bold values are the best metric values.*

**FIGURE 6:**
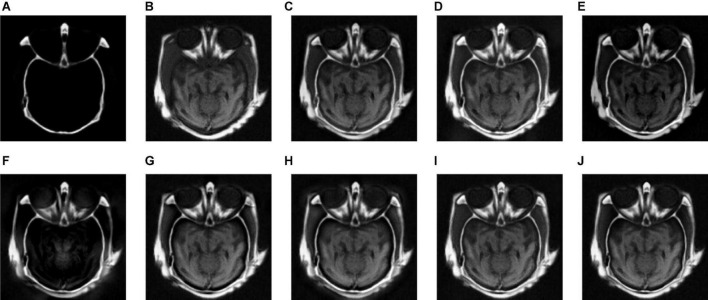
The fusion results of Group (a). **(A,B)** The source images. **(C–J)** The fusion results of GFF, CNN, CSMCA, NSCT-PCNN, NSCT-PCLLE, NSST-PAPCNN, RGF-CNM, and the proposed algorithm.

**FIGURE 7:**
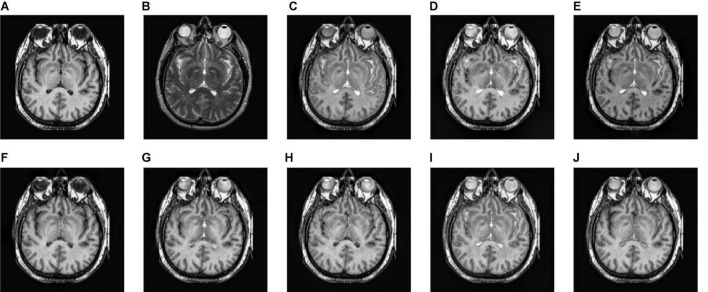
The fusion results of Group (b). **(A,B)** The source images. **(C–J)** The fusion results of GFF, CNN, CSMCA, NSCT-PCNN, NSCT-PCLLE, NSST-PAPCNN, RGF-CNM, and the proposed algorithm.

**FIGURE 8:**
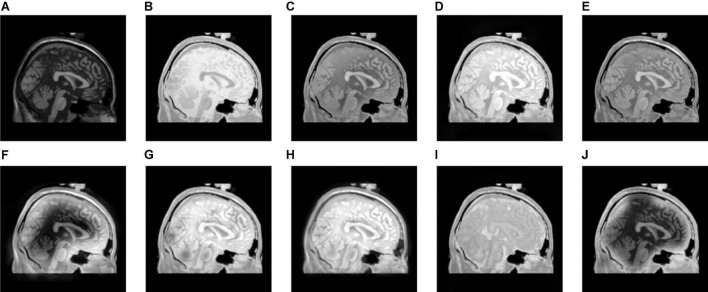
The fusion results of Group (c). **(A,B)** The source images. **(C–J)** The fusion results of GFF, CNN, CSMCA, NSCT-PCNN, NSCT-PCLLE, NSST-PAPCNN, RGF-CNM, and the proposed algorithm.

**FIGURE 9:**
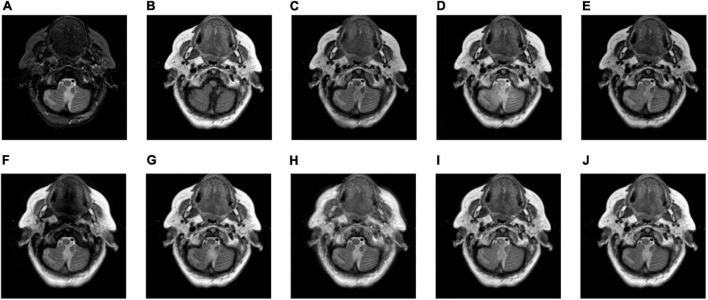
The fusion results of Group (d). **(A,B)** The source images. **(C–J)** The fusion results of GFF, CNN, CSMCA, NSCT-PCNN, NSCT-PCLLE, NSST-PAPCNN, RGF-CNM, and the proposed algorithm.

**FIGURE 10:**
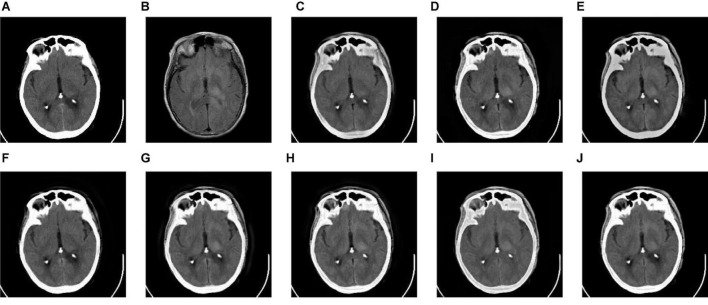
The fusion results of Group (e). **(A,B)** The source images. **(C–J)** The fusion results of GFF, CNN, CSMCA, NSCT-PCNN, NSCT-PCLLE, NSST-PAPCNN, RGF-CNM, and the proposed algorithm.

We tested all the methods in the test image sets in Figure 3 and the fused images are displayed in Figures 6–10. Figure 6 shows the fusion results of Group (a). Figures 6A,B show the source images. And Figures 6C–J are the fusion results of GFF, CNN, CSMCA, NSCT-PCNN, NSCT-PCLLE, NSST-PAPCNN, RGF-CNM, and the proposed algorithm, respectively.

Figure 6 shows that the fused image of our algorithm has a higher contrast, shows more structural information and textural information, and introduces less useless information among these algorithms. And the edges of Figure 6C are not clear enough, and GFF lost some structural information. Figure 6D retains the spatial information of source images, but some edges are lost. Figure 6E has low contrast, and the fusion effect is not ideal. Figure 6F shows that the CSMCA does not get a good fused image, and cannot be able to retain useful information effectively. Although Figure 6G retains more useful information of source images, but a small number of artifacts are produced. There are unwanted artifacts in Figure 6H. The contrast in Figure 6I is high, but the edges are blurred.

Table 2 shows the objective evaluation results of Group (a) for all test algorithms. As seen in Table 2, our fusion algorithm has the highest values on MI, QAB/F, SSIM, Piella, and UIQI. The maximum values of MI and QAB/F indicate that our algorithm retains more useful information and edges.

**TABLE 2:** Objective evaluation results of Group (a).

	MI	Q^AB/F^	SSIM	VIFF	UIQI	Q	Q_*W*_	Q_*E*_	R${}_{Q}^{A\,B/F}$
GFF	3.4313	0.7789	0.4865	0.4865	0.4493	0.8147	0.7889	0.7843	0.3824
CNN	3.0757	0.7683	0.4764	**0.5453**	0.4451	0.7945	0.7805	0.7819	0.3810
CSMCA	2.5864	0.7280	0.4388	0.4752	0.3414	0.5721	0.6024	0.6171	0.3715
NSCT-PCNN	1.2824	0.5764	0.3990	0.3313	0.2678	0.4257	0.4381	0.4443	0.3441
NSCT-PCLLE	3.2906	0.7325	0.4861	0.4625	0.4512	0.8077	0.7878	0.7799	0.3734
NSST-PAPCNN	2.4665	0.6859	0.4637	0.4559	0.3950	0.6996	0.7030	0.5812	0.3683
RGF-CNM	4.1236	0.7808	0.4986	0.5105	0.4760	0.8475	0.8231	0.7947	**0.3844**
DWTRP-CNN	**4.3328**	**0.7869**	**0.4990**	0.4684	**0.4770**	**0.8636**	**0.8300**	**0.8185**	0.3839

*The bold values are the best metric values.*

In Table 2, the highest indicators of SSIM and Piella indicate the highest structural similarity between the fused image and the source images obtained by our algorithm. The maximum value of UIQI indicates that the correlation, brightness, and contrast of the fused image and the source images obtained by our algorithm are closest. Although the VIFF of our algorithm is not the highest, from the visual effects of Figure 6, our fusion algorithm is the best. In all comparison algorithms, the VIFF value of CNN is the highest, which is better than our method. However, the other seven evaluation indexes are lower than our fusion algorithm, and the value of MI is far from ours, which indicates that CNN is not ideal in preserving the information of source images. Therefore, our algorithm is an effective medical image fusion algorithm.

Figure 7 gives the fusion results of Group (b). In Figure 7, the contrasts of Figures 7C,E are low, and the image in Figure 7F loses more useful information. In addition, Figures 7F,I produce different degrees of blocking effects and artifacts. Figures 7D,G,H fuse the two source medical images well, but still lose a small part of detailed and textural information, and do not get the best visual effects. Compared to the contrasting algorithms, the fused image of our algorithm has the best visual effect.

Table 3 shows the objective evaluation results of Group (b) for all test algorithms. Table 3 shows that the proposed algorithm gives the best objective evaluation results except VIFF and QW. It indicates that the proposed algorithm retains the most useful information and edges, the structural similarity of fused image and source images is the highest, and the correlation, brightness, and contrast are the closest. In other contrast algorithms, the values of SSIM, VIFF, and QW of NSST-PAPCNN fusion algorithm are the highest, but other evaluation indexes are lower than the proposed algorithm, especially MI and QAB/F. From the visual effects and objective evaluation, the proposed algorithm achieves a satisfying fusion effect.

**TABLE 3:** Objective evaluation results of Group (b).

	MI	Q^AB/F^	SSIM	VIFF	UIQI	Q	Q_*W*_	Q_*E*_	R${}_{Q}^{A\,B/F}$
GFF	4.1027	0.6057	0.6671	0.5213	0.4734	0.5022	0.5077	0.5009	0.3544
CNN	3.8871	0.5855	0.6433	0.5775	0.4145	0.4639	0.5096	0.4884	0.3502
CSMCA	3.6764	0.6062	0.6441	0.5273	0.3774	0.3818	0.4771	0.4634	0.3525
NSCT-PCNN	4.4815	0.5787	0.6532	0.5104	0.4552	0.4019	0.4830	0.4586	0.3506
NSCT-PCLLE	4.1448	0.5618	0.6655	0.5708	0.4688	0.5022	0.5134	0.4859	0.3466
NSST-PAPCNN	3.8906	0.5228	**0.6814**	**0.5931**	0.4576	0.5028	**0.5374**	0.4858	0.3420
RGF-CNM	4.4565	0.6130	0.6800	0.5804	0.4870	0.4536	0.5119	0.4822	0.3570
DWTRP-CNN	**4.9866**	**0.8185**	**0.6814**	0.5584	**0.5634**	**0.5551**	0.5317	**0.5113**	**0.3575**

*The bold values are the best metric values.*

Figures 8–10 show the fusion results of groups (c), (d), and (e). Similar to Figures 6, 7, the proposed method retains more structural and detailed information of the input medical images, has higher contrast and brightness, and introduces less useless information, such as blocking effects and artifacts from the fusion results of Figures 8C–J, 9C–J, 10C–J.

From the comprehensive view of the objective indicators in Tables 4–6, the proposed algorithm has higher objective evaluation indicators than other algorithms. In addition, Table 7 shows the running times of these algorithms. Compared to others, the running time of our algorithm is also relatively competitive. Therefore, the proposed algorithm is an effective and robust medical image fusion algorithm.

**TABLE 4:** Objective evaluation results of Group (c).

	MI	Q^AB/F^	SSIM	VIFF	UIQI	Q	Q_*W*_	Q_*E*_	R${}_{Q}^{A\,B/F}$
GFF	2.9724	0.6567	0.6598	0.6146	0.4472	0.2462	0.3401	0.4045	0.3560
CNN	3.5573	0.6454	0.5050	**0.7817**	0.2930	0.2098	0.2897	0.3700	0.3528
CSMCA	3.0675	0.6509	0.6752	0.6266	0.5557	0.1904	0.3211	0.3912	0.3513
NSCT-PCNN	2.8244	0.5922	0.5382	0.6118	0.3777	0.1840	0.3243	0.3713	0.3396
NSCT-PCLLE	3.3006	0.6111	0.6392	0.7402	0.4883	0.2332	0.2917	0.3623	0.3430
NSST-PAPCNN	3.1406	0.5525	0.5564	0.6229	0.3141	0.1664	0.2522	0.3114	0.3371
RGF-CNM	3.4611	0.5414	0.6412	0.6863	0.3720	0.2323	0.3082	0.2920	0.3401
DWTRP-CNN	**3.8684**	**0.8569**	**0.6854**	0.6645	**0.6088**	**0.3040**	**0.3986**	**0.4272**	**0.3567**

*The bold values are the best metric values.*

**TABLE 5:** Objective evaluation results of Group (d).

	MI	Q^AB/F^	SSIM	VIFF	UIQI	Q	Q_*W*_	Q_*E*_	R${}_{Q}^{A\,B/F}$
GFF	3.2966	0.5892	0.6982	0.6020	0.5022	0.4847	0.5482	0.4916	0.3398
CNN	3.6331	0.5915	0.7038	0.7405	0.5203	0.3218	0.5248	0.4476	0.3383
CSMCA	3.3221	0.6079	0.7131	0.6405	0.5570	0.483	0.5159	0.4493	0.3381
NSCT-PCNN	2.9174	0.4276	0.6266	0.6606	0.3989	0.3738	0.5048	0.4461	0.3150
NSCT-PCLLE	3.8238	0.5714	0.7303	0.7731	0.5807	0.5331	0.5754	0.5006	0.3354
NSST-PAPCNN	3.2064	0.4081	0.6527	0.6169	0.4294	0.4031	0.5250	0.4640	0.3193
RGF-CNM	4.1837	0.6338	0.7318	0.7436	0.5504	0.3717	0.5494	0.4725	0.3464
DWTRP-CNN	**4.5808**	**0.8423**	**0.7320**	**0.7801**	**0.6038**	**0.5570**	**0.5837**	**0.5266**	**0.3473**

*The bold values are the best metric values.*

**TABLE 6:** Objective evaluation results of Group (e).

	MI	Q^AB/F^	SSIM	VIFF	UIQI	Q	Q_*W*_	Q_*E*_	R${}_{Q}^{A\,B/F}$
GFF	3.4014	0.6171	0.7709	0.4688	0.6538	0.2954	0.4988	0.4935	0.3538
CNN	3.3405	0.6232	0.6745	0.5078	0.3448	0.2977	0.5127	0.5081	0.3576
CSMCA	3.1097	0.5933	0.7568	0.4745	0.5622	0.2731	0.4830	0.4840	0.3402
NSCT-PCNN	3.7688	0.5683	0.7257	0.4157	0.4307	0.2818	0.4623	0.4854	0.3398
NSCT-PCLLE	3.3127	0.5841	0.7153	0.4892	0.5427	0.3005	0.5002	0.4884	0.3474
NSST-PAPCNN	3.2213	0.5902	0.7180	0.5031	0.5496	0.3116	0.5207	0.5058	0.3546
RGF-CNM	3.5551	0.6131	0.7786	0.5206	0.5140	0.3247	**0.5352**	0.4896	**0.3597**
DWTRP-CNN	**3.7814**	**0.6326**	**0.7864**	**0.5316**	**0.6764**	**0.3352**	0.5286	**0.5319**	0.3556

*The bold values are the best metric values.*

**TABLE 7:** The running times of all fusion algorithms.

Time(s)	Group (a)	Group (b)	Group (c)	Group (d)	Group (e)
GFF	**0.0550**	**0.0346**	**0.0363**	**0.0449**	**0.0661**
CNN	10.3217	10.6671	10.3465	10.7059	10.2416
CSMCA	85.1345	84.7032	85.8902	86.4450	85.4226
NSCT-PCNN	39.5787	39.9496	39.6413	39.6515	39.7292
NSCT-PCLLE	2.1394	1.7340	1.7377	1.7313	1.7528
NSST-PAPCNN	4.0287	4.0262	3.9829	4.5434	4.1555
RGF-CNM	34.2282	34.6446	33.4955	35.2557	33.6108
DWTRP-CNN	11.3148	11.5726	10.8541	11.5949	11.1146

*The bold values are the best metric values.*

**FIGURE 11:**
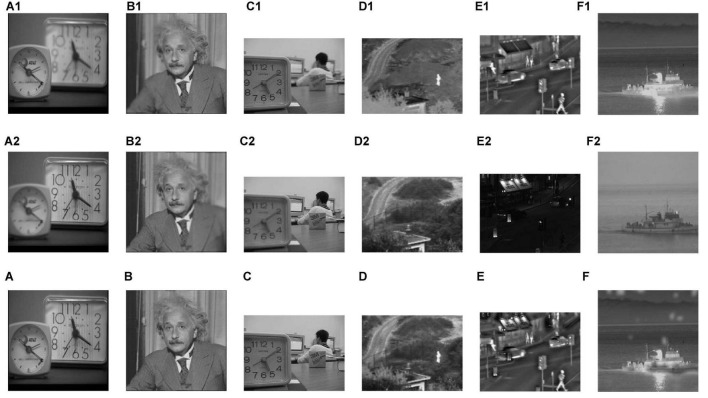
The fusion results of multi-focus image fusion and infrared and visible image fusion. **(C)** The fusion result without CNN. **(D)** The fusion result with CNN. **(A–F)** The fused images. **(A1–F1)** The one source images of each group. **(A2–F2)** The other one source images.

In addition, we applied the proposed algorithm to multi-focus image fusion and infrared and visible image fusion. In Figures 11A1–F1 are the one source images of each group. Figures 11A2–F2 are the other one source images. Figures 11A–F are the fused images. As shown in Figure 11, the proposed fusion algorithm is also effective in multi-focus image fusion and infrared and visible image fusion.

## Conclusion

In this article, we proposed a two-scale multimodal medical image fusion algorithm based on structure preservation. The proposed algorithm decomposes the source images by two-scale decomposition, which fully uses the multi-scale information of the images. Our algorithm also adopts the structure preservation characteristic of the iterative joint bilateral filter and applies CNN in medical image fusion. From the visual effects and objective measures, the contrast experiments show that the proposed algorithm has better performance than the compared algorithms. However, the speed of the proposed algorithm is not ideal. In future, the computational speed of the proposed algorithm will be optimized for practical application in clinical practice.

## Data Availability Statement

Publicly available datasets were analyzed in this study. This data can be found here: http://www.med.harvard.edu/aanlib/home.html.

## Ethics Statement

Written informed consent was obtained from the individual(s) for the publication of any potentially identifiable images or data included in this article.

## Author Contributions

LY performed the computer simulations. SL, XS, and JZ analyzed the data. SL wrote the original draft. XS and Y-DZ revised and edited the manuscript. MW polished the manuscript. All authors approved the submitted version.

## Conflict of Interest

The authors declare that the research was conducted in the absence of any commercial or financial relationships that could be construed as a potential conflict of interest.

## Publisher’s Note

All claims expressed in this article are solely those of the authors and do not necessarily represent those of their affiliated organizations, or those of the publisher, the editors and the reviewers. Any product that may be evaluated in this article, or claim that may be made by its manufacturer, is not guaranteed or endorsed by the publisher.
